# Metformin Increases Sensitivity of Pancreatic Cancer Cells to Gemcitabine by Reducing CD133^+^ Cell Populations and Suppressing ERK/P70S6K Signaling

**DOI:** 10.1038/srep14404

**Published:** 2015-09-22

**Authors:** Xinqun Chai, Hongpeng Chu, Xuan Yang, Yuanpu Meng, Pengfei Shi, Shanmiao Gou

**Affiliations:** 1 Department of Hepatobiliary Surgery, Union Hospital, Tongji Medical College, Huazhong University of Science and Technology; 2 Department of Breast and Thyroid Surgery, Central Hospital of Wuhan; 3 Pancreatic Disease Institute, Union Hospital, Tongji Medical College, Huazhong University of Science and Technology.

## Abstract

The prognosis of pancreatic cancer remains dismal, with little advance in chemotherapy because of its high frequency of chemoresistance. Metformin is widely used to treat type II diabetes, and was shown recently to inhibit pancreatic cancer stem cell proliferation. In the present study, we investigated the role of metformin in chemoresistance of pancreatic cancer cells to gemcitabine, and its possible cellular and molecular mechanisms. Metformin increases sensitivity of pancreatic cancer cells to gemcitabine. The mechanism involves, at least in part, the inhibition of CD133^+^ cells proliferation and suppression of P70S6K signaling activation via inhibition of ERK phosphorylation. Studies of primary tumor samples revealed a relationship between P70S6K signaling activation and the malignancy of pancreatic cancer. Analysis of clinical data revealed a trend of the benefit of metformin for pancreatic cancer patients with diabetes. The results suggested that metformin has a potential clinical use in overcoming chemoresistance of pancreatic cancer.

Pancreatic cancer is among the most aggressive of solid malignancies^1^,^2^,^3^,^4^. Each year, 45,220 patients are newly diagnosed with the disease, resulting in 38,460 deaths per annum in the United States, and making pancreatic cancer the fourth leading cause of cancer related death in both males and females^5^.

Gemcitabine was recommended by the National Comprehensive Cancer Network (NCCN) guidelines as the first first-line drug for chemotherapy of pancreatic cancer^6^; however, its efficacy is dismal^7^,^8^, which is partly because of the chemoresistance of pancreatic cells. Recently studies showed that a subpopulation of pancreatic cells that expressed CD133^+^ has characteristics of cancer stem cells, and these cells were hypothesized to play a key role in chemoresistance^9^,^10^,^11^. In our previous study, we showed that metformin selectively inhibited the proliferation and invasion of the CD133^+^ subpopulation of pancreatic cancer cells^12^. Thus, metformin may have the capacity to attenuate the chemoresistance of pancreatic cancer cells to gemcitabine.

Here, we showed that metformin enhanced the capacity of gemcitabine to inhibit the proliferation and invasion of pancreatic cancer cells, by inhibiting the proliferation of CD133^+^ cell populations. Phosphorylation of P70S6K, one of the two major direct targets of mTOR signaling^13^, and the anticancer actions of mTOR inhibitors are mediated primarily through P70S6K inhibition^14^. The inhibition of P70S6K signaling activation by attenuating ERK phosphorylation, which is associated with the malignancy of pancreatic cancer, is thought to contribute to this effect.

## Results

### CD133^+^ pancreatic cancer cells have a higher capacity to resist gemcitabine

**Figure 1:**
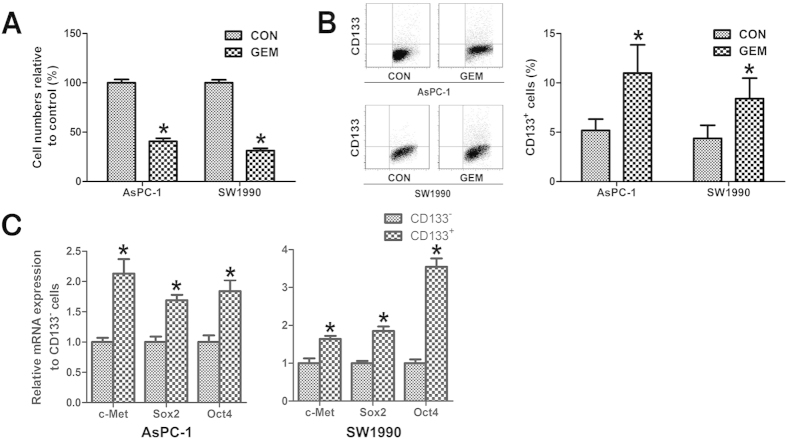
CD133^+^ pancreatic cancer cells had a higher capacity to resist gemcitabine. (**A**) AsPC-1 and SW1990 pancreatic cancer cells were treated with 300 nM gemcitabine for 48 h, and the numbers of viable cells were determined by a CCK-8 assay. The results are presented as the proportion of viable cells relative to the control. Significant inhibition of pancreatic cancer cells proliferation was observed in gemcitabine treated cells. (**B**) AsPC-1 and SW1990 pancreatic cancer cells were treated with 300 nM gemcitabine for 48 h, and the proportion of CD133^+^ cells was determined by flow cytometry. The proportion of CD133^+^ cells was significantly higher in gemcitabine treated group than in the control group. (**C**) CD133^−^ and CD133^+^ cells were isolated from AsPC-1 and SW1990 pancreatic cancer cells. The results are presented as the relative mRNA expression to CD133^−^ cells. The *c-Met*, *Sox2 and Oct4* mRNA expression in CD133^+^ cells were significantly higher than that in CD133^−^ cells. CON, control; GEM, gemcitabine; MET, metformin. ^*^P < 0.05.

To investigate the effect of gemcitabine on the proliferation of different subpopulations of pancreatic cancer cells, we conducted CCK-8 assays and flow cytometry assay using AsPC-1 and SW1990 cells. The cells were treated with 300 nM gemcitabine for 48 h. As shown in Fig. 1A,B and Supplementary Table S1, gemcitabine treatment resulted in significant inhibition of cell proliferation of both AsPC-1 and SW1990 cells, with an increase of the proportion of CD133^+^ cells, which suggested that CD133^+^ cells have a higher capacity to resist gemcitabine.

We next measured the relative mRNA levels of pluripotency marker genes of cancer stem cells, *c-Met*, *Sox2 and Oct4*, in CD133^−^ and CD133^+^ pancreatic cancer cells. As shown in Fig. 1C, the *c-Met*, *Sox2 and Oct4* mRNA expressions in CD133^+^ cells were significantly higher than those in CD133^−^ cells, which suggested that CD133^+^ cells have characteristics of cancer stem cells.

The CD24^+^CD44^+^ESA^+^ cells, which was also documented to be with characteristics of cancer stem cells, didn’t show high capacity to resist gemcitabine (Supplementary Figure S1).

### Metformin enhanced the sensitivity of pancreatic cancer cells to gemcitabine

**Figure 2:**
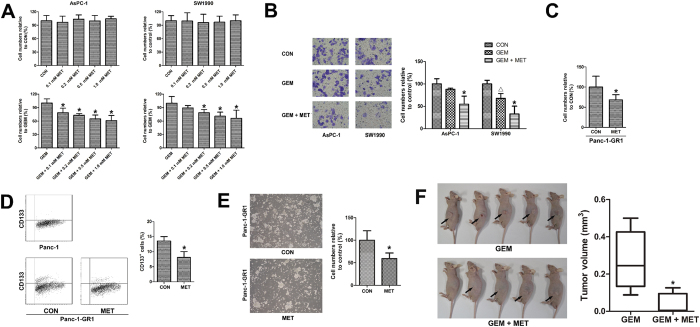
Metformin enhanced the sensitivity of pancreatic cancer stem cells to gemcitabine. (**A**) Pancreatic cancer cells were treated with different concentrations of metformin only or with 300 nM gemcitabine for 48 h, and the numbers of viable cells were determined by a trypan blue assay. The results are presented as the proportion of viable cells relative to the control or gemcitabine group. No difference was observed between cells treated with metformin and controls. The numbers of viable cells were reduced in a dose-dependent manner when metformin was combined with gemcitabine. ^*^P_*GEM vs GEM+MET*_ < 0.05. (**B**) The effect of metformin on invasion by pancreatic cancer. Pancreatic cancer cells were incubated with 300 nM gemcitabine only or with 1 mM metformin for 24 h, and cell invasion was determined by a Transwell assay. Metformin reduced the invasion of pancreatic cancer cells when combined with gemcitabine. ^△^*P*_*GEM vs CON*_ < 0.05, ^*^P_*GEM vs GEM+MET*_ < 0.05. (**C**) gemcitabine-resistant Panc-1-GR1 cells were treated with 1 mM metformin for 48 h, and numbers of viable cells were determined by a trypan blue assay. The results are presented as the proportion of viable cells relative to the control group. The viable cells were reduced in the metformin group. ^*^P < 0.05. (**D**) the proportions of CD133^+^ cells were determined by flow cytometry. The proportion of CD133^+^ cells was significantly higher in gemcitabine-resistant Panc-1-GR1 cells than in Panc-1 cells. The proportions of CD133^+^ cells in Panc-1-GR1 cells were significantly reduced by 1 mM metformin treatment for 96 h. ^*^P < 0.05. (**E**) gemcitabine-resistant Panc-1-GR1 cells were cultured in a cancer stem cell spheres culture system and treated with 1 mM metformin for 14 days. The formation of spheres was significantly reduced by metformin. ^*^P < 0.05. (**F**) For mice treated with metformin, 800 mg/L of metformin was diluted in the drinking water of *nu*/*nu* mice. Xenografts from mice treated with oral metformin were much smaller than those from untreated mice 4 weeks after the implantation. ^*^P < 0.05. CON, control; GEM, gemcitabine; MET, metformin.

To investigate the effect of metformin on the sensitivity of pancreatic cancer cells to gemcitabine, we conducted trypan blue assays and Transwell invasion assays using AsPC-1 and SW1990 cells. Figure 2A shows that metformin alone (0.1 to 1 mM) did not inhibit the survival of pancreatic cancer cells. However, when combined with gemcitabine, metformin inhibited the survival of pancreatic cancer cells. Figure 2B shows that metformin enhanced the capacity of gemcitabine to inhibit invasion of pancreatic cancer cells.

Trypan blue assays, flow cytometry and sphere culture of Panc-1-GR1 cells were conducted to investigate the role of metformin on gemcitabine-resistant pancreatic cancer cells. As shown in Fig. 2C, 1 mM metformin significantly inhibited the proliferation of gemcitabine-resistant pancreatic cancer cells. Figure 2D shows that the proportion of CD133^+^ cells was much higher in Panc-1-GR1 cells than in Panc-1 cells, suggesting the enrichment of pancreatic cancer stem cells. After treatment with 1 mM metformin, the proportion of CD133^+^ cells decreased significantly in Panc-1-GR1 cells. Figure 2E shows the sphere culture of Panc-1-GR1 cells. Metformin at 1 mM significantly inhibited the formation of cancer stem cell spheres.

To investigate the effect of metformin on pancreatic cancer *in vivo*, xenograft experiments using *nu*/*nu* mice were conducted. Mice were injected subcutaneously with 1 × 10^7^ Panc-1-GR1 pancreatic cancer cells on their left flank. For mice treated with metformin, the amount of drug diluted in their drinking water was equivalent to a human dose of 20 mg/kg by normalization to surface area. Both the gemcitabine treatment and the metformin treatment began at the time of injection with the pancreatic cancer cells. Mice were sacrificed 4 weeks after they were injected with pancreatic cancer cells. The growth of the pancreatic cancer xenografts was significantly inhibited by metformin treatment (Fig. 2F).

### Malignancy of pancreatic cancer is associated with the activation of P70S6K signaling

**Figure 3:**
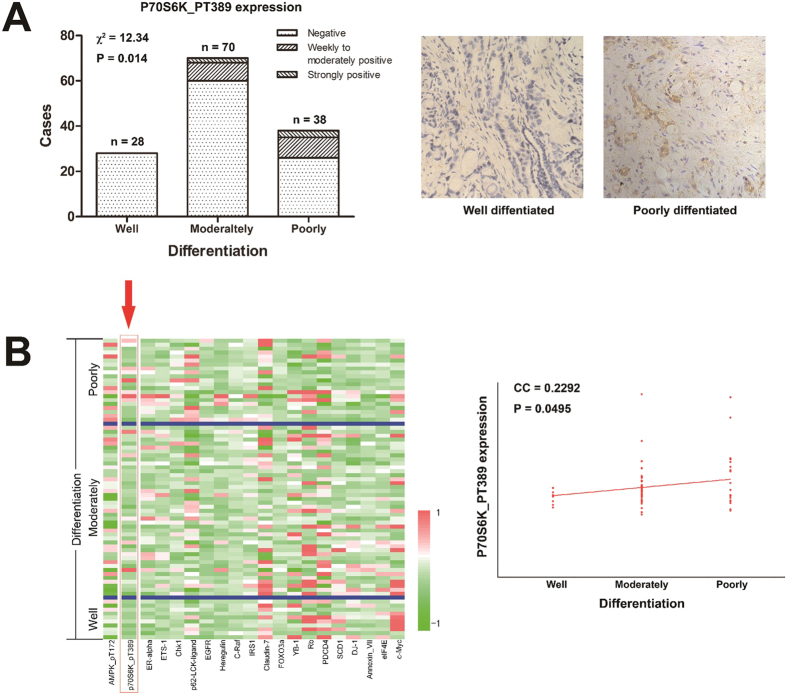
Malignancy of pancreatic cancer correlates with the activation of P70S6K signaling. (**A**) expression of phospho-P70S6K in pancreatic cancer from the Union Hospital using immunohistochemistry. Tumor samples were grouped by histological grade: well differentiated (n = 28), moderately differentiated (n = 70) and poorly differentiated (n = 38). (**B**) Heatmap of 20 proteins with the smallest *P* values (0.0361 to 0.1914) that correlated with the histological grade of pancreatic cancer using RPPA. The RPPA data were downloaded from TCGA Research Network. Tumor samples were grouped by histologic grade: well differentiated (n = 10), moderately differentiated (n = 43) and poorly differentiated (n = 21). *P* values of 4 proteins were less than 0.05: *P*_c-Myc_ = 0.0361, *P*_phospho-AMPK_ = 0.0425, *P*_phospho-P70S6K_ = 0.0495 and *P*_eIF4E_ = 0.0495. The phosphor-P70S6K expression is marked by an arrow. The right column showed dot map of phospho-P70S6K expression in pancreatic cancer with different histologic grade using RPPA. CC, correlation coefficient.

Immunohistochemistry and reverse phase protein array (RPPA) analysis were performed to analyze the correlation between the histological grade of pancreatic cancer and phospho-P70S6K (p70 S6 Kinase, Thr389). Immunohistochemistry of phosphor-P70S6K was conducted on 136 samples of pancreatic cancer from Union Hospital, Wuhan, of which 28 were well differentiated, 70 were moderately differentiated and 38 were poorly differentiated. Immunohistochemistry revealed that high expression of phospho-P70S6K was associated with high histological grade of pancreatic cancer (Fig. 3A).

The RPPA data for pancreatic cancer were downloaded from TCGA Research Network. RPPA analyses were conducted from tumor protein lysates of 74 tumor samples, of which 10 were well differentiated, 43 were moderately differentiated and 21 were poorly differentiated. Of the 193 proteins analyzed, only four proteins were significantly associated with the histological grade of pancreatic cancer (*P* < 0.05); *i.e.* c-Myc, phospho-AMPK, eIF4E and phospho-P70S6K (Supplementary Table S2). Figure 3B shows the heatmap of 20 proteins with *P* values from 0.0361 to 0.1914, and tumor samples of high histological grade with high expression of phospho-P70S6K. The *P* values of the other 173 proteins ranged from 0.1992 to 0.9994. These results suggested the activation of P70S6K signaling in highly malignant pancreatic cancer.

### Metformin inhibited phosphorylation of P70S6K by inhibition of ERK1/2 activation

**Figure 4:**
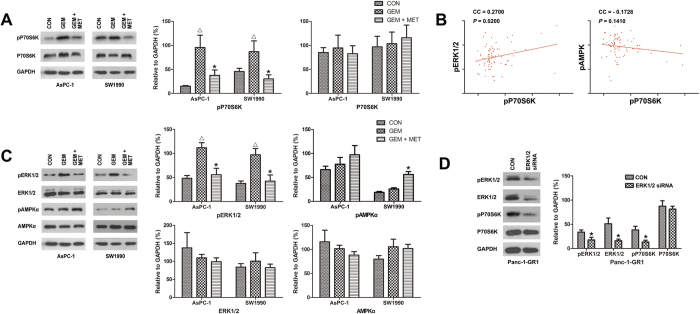
Metformin inhibited phosphorylation of P70S6K by inhibition of ERK1/2 activation. (**A**) pancreatic cancer cells were treated with 300 nM gemcitabine only or with 1 mM metformin for 4 hours. Expressions of proteins were evaluated by western blotting and the results were quantified using ImageJ V.1.46r (National Institutes of Health). Significant decreases in phospho-P70S6K expression were observed in the metformin treated cells. ^△^*P*_*GEM vs. CON*_ < 0.05, ^*^P_*GEM vs. GEM+MET*_ < 0.05. (**B**) The relationship between phospho-P70S6K and phospho-ERK1/2 or phospho-AMPKα using reverse phase protein array (RPPA) analysis (n = 74). The expression of phospho-P70S6K was associated with phospho-ERK1/2 (*P* = 0.02), but not with phospho-AMPKα (*P* = 0.14). (**C**) pancreatic cancer cells were treated with 300 nM gemcitabine only or with 1 mM metformin for 4 hours. Expression of proteins were evaluated by western blotting and the results were quantified using ImageJ V.1.46r. Significant increase in phospho-ERK1/2 expression were observed in the gemcitabine treated cells, which was inhibited by treatment with metformin. A significant increase in phospho-AMPKα expression was observed in the metformin treated SW1990 cells, but not in AsPC-1 cells. ^△^*P*_*GEM vs. CON*_ < 0.05, ^*^P_*GEM vs. GEM+MET*_ < 0.05. (**D**) gemcitabine-resistant Panc-1-GR1 cells were transfected with an ERK1/2 siRNA. Expression of proteins were evaluated by western blotting 72 hours after transfection, and the results were quantified using ImageJ V.1.46r. Significant decreases in phospho-P70S6K expression were observed in the ERK1/2 siRNA cells. ^*^P < 0.05. CON, control; GEM, gemcitabine; MET, metformin; CC, correlation coefficient.

As shown in Fig. 4A, gemcitabine alone significantly increased the phosphorylation of P70S6K in both AsPC-1 and SW1990 pancreatic cells. When combined with metformin, the phosphorylation of P70S6K induced by gemcitabine was significantly reduced.

RPPA analysis of 74 pancreatic cancer samples was conducted to determine the relationship between P70S6K phosphorylation and ERK1/2 or AMPK phosphorylation. As shown in Fig. 4D, P70S6K phosphorylation was associated with ERK1/2 phosphorylation (P = 0.02); however, P70S6K phosphorylation did not correlate with AMPK phosphorylation in pancreatic cancer.

To identify possible molecular determinants of the effects of metformin on phosphorylation of P70S6K, we evaluated the activation of AMPK and ERK, which might be involved in these effects. As shown in Fig. 4B, gemcitabine alone significantly increased the phosphorylation of ERK1/2 in both AsPC-1 and SW1990 pancreatic cells. AMPK phosphorylation did not increase or decrease in AsPC-1 cells or in SW1990 cells. When combined with metformin, the phosphorylation of ERK1/2 induced by gemcitabine was significantly reduced, while phosphorylation of AMPK was enhanced in SW1990 cells, but not in AsPC-1 cells.

We next transfected gemcitabine-resistant Panc-1-GR1 pancreatic cancer cells with siRNA targeting ERK1/2. As shown in Fig. 4C, ERK1/2 knockdown inhibited the phosphorylation of P70S6K.

### ERK1/2 knockdown partly mimics the actions of metformin on gemcitabine-resistant pancreatic cancer cells

**Figure 5:**
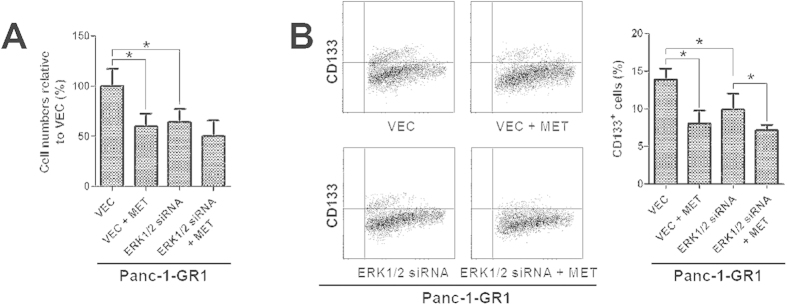
ERK1/2 knockdown partly mimics the actions of metformin on gemcitabine-resistant pancreatic cancer cells. (**A**) gemcitabine-resistant Panc-1-GR1 cells were transfected with an ERK1/2 siRNA. 48 h after the transfection, cells were treated with 1 mM metformin for 48 h, and numbers of viable cells were determined by a trypan blue assay. The results are presented as the proportion of viable cells relative to the vector group. The viable cells were reduced in the ERK1/2 siRNA group. When treated with metformin, the reduction of viable cells in ERK1/2 knockdown cells was attenuated compared with that in vector cells. ^*^P < 0.05. (**B**) gemcitabine-resistant Panc-1-GR1 cells were transfected with an ERK1/2 siRNA. 48 h after the transfection, cells were treated with 1 mM metformin for 96 h, and the proportions of CD133^+^ cells were determined by flow cytometry. The proportions of CD133^+^ cells in Panc-1-GR1 cells were significantly reduced by ERK1/2 knockdown. When treated with metformin, the reduction of CD133^+^ cells in ERK1/2 knockdown cells was attenuated compared with that in vector cells. ^*^P < 0.05. VEC, vector; MET, metformin.

To investigate the role ERK1/2 phosphorylation reduction for the actions of metformin on gemcitabine-resistant pancreatic cancer cells, gemcitabine-resistant Panc-1-GR1 pancreatic cancer cells were transfected with siRNA targeting ERK1/2. As shown in Fig. 5A,B, ERK1/2 knockdown mimics the actions of metformin on gemcitabine-resistant pancreatic cancer cells that inhibiting cell proliferation and decreasing CD133^+^ cell proportion. When treated with metformin, the actions were attenuated in ERK1/2 knockdown cells. The metformin at 1mM didn’t inhibit cell proliferation significantly in ERK1/2 knockdown cells. Although metformin at 1 mM decreased the proportion of CD133^+^ cells significantly in ERK1/2 knockdown cells, the decrease was less than that in cells transfected with vector.

### Metformin showed a trend of improved prognosis of patients receiving gemcitabine

**Figure 6:**
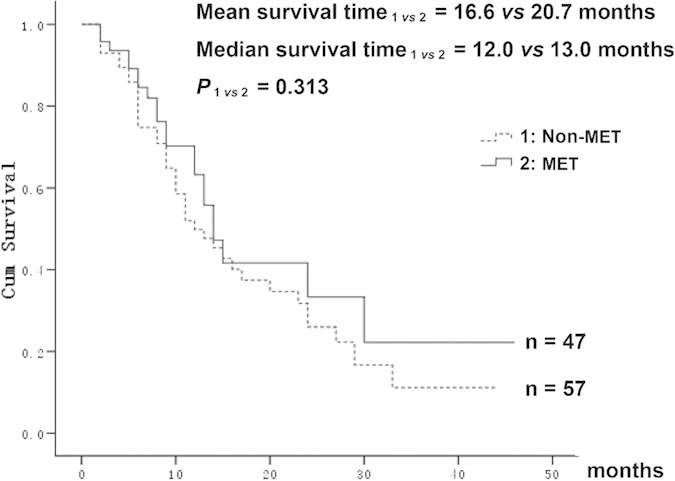
Metformin improved the prognosis of patients. Survival curve of patients with diabetes after radical pancreatic cancer resection and receiving gemcitabine treatment. Patients receiving metformin treatment showed a trend of better prognosis. CON, control; GEM, gemcitabine; MET, metformin.

One hundred and four patients with diabetes that received gemcitabine treatment after radical resection of pancreatic cancer in Union Hospital, Wuhan, China from Jun 1^st^, 2009 to Jun 20^th^, 2014 were followed up. Of these patients, 47 received metformin treatment, while the other 57 did not. As shown in Fig. 6, the median overall survival time was 12.0 months for the non-metformin group, and 13.0 months for the metformin group. The mean survival time was 16.6 months for the non-metformin group, and 20.7 months for the metformin group. The metformin group showed a trend of having better survival compared with the non-metformin group, although the difference between the two groups was not statistically significant.

## Discussion

The findings of the present study indicated that metformin increases sensitivity of pancreatic cancer cells to gemcitabine and may benefit patients with pancreatic cancer that receive gemcitabine after pancreatic cancer resection. The analysis of the cellular and molecular mechanism suggested that the reduction of CD133^+^ cell populations and inhibition of P70S6K signaling activation play important roles.

Treating pancreatic cancer is difficult because of its high frequency of recurrence, metastasis and resistance to chemotherapies. Gemcitabine is the first-line drug for chemotherapy of pancreatic cancer, according to the NCCN clinical practice guidelines in oncology; however, the 5-year survival was only about 10% after pancreatic cancer resection in patients who received gemcitabine treatment for local recurrence and distant metastasis^15^. Recent studies suggested that cancer stem cells are the culprits in tumor relapse and the cause of chemotherapy failures^16^,^17^,^18^. To overcome chemoresistance and improve the outcome of pancreatic cancer, pancreatic cancer stem cells should be targeted effectively. Recently, metformin was reported to target cancer stem cells in various cancer types^19^,^20^. Metformin is the most frequently prescribed antidiabetic drug for type II diabetes^21^. Diabetes and pancreatic cancer have a complex relationship. Long-term diabetes is a risk factor for pancreatic cancer. On the other hand, patients with pancreatic cancer are often diagnosed subsequently with diabetes. The prevalence of diabetes or impaired glucose tolerance in patients of pancreatic cancer can be up to 80%^22^. Interestingly, metformin must be actively transported into cells by the transmembrane protein organic cation transporter 1 and 2, and these proteins are highly expressed in pancreatic cancer cells^23^. A case control study by Li *et al.* focusing on the effect of antidiabetic therapies on the risk of pancreatic cancer, demonstrated that metformin significantly decreased the risk of pancreatic cancer, with an odds ratio of 0.38^24^. We have documented previously that metformin selectively inhibits pancreatic cancer stem cell proliferation and has anticancer action^25^. Thus, we hypothesized that metformin may increase the sensitivity of pancreatic cancer to gemcitabine by targeting pancreatic cancer stem cells.

Initially, we analyzed whether CD133^+^ pancreatic cells, which have characteristics of cancer stem cells, are resistant to gemcitabine treatment. A combination analysis of total cell viability and CD133^+^ cell proportion indicated that CD133^+^ pancreatic cancer cells are resistant to gemcitabine treatment. We then analyzed the role of metformin on gemcitabine resistance in pancreatic cancer. Metformin alone at 1 mM had almost no effect on the proliferation of pancreatic cancer cells, but did inhibit the proliferation of pancreatic cancer cells significantly when combined with gemcitabine. Furthermore, metformin significantly enhanced the capacity of gemcitabine to inhibit the invasion of pancreatic cancer cells.

To further investigate the role of metformin on the stemness of pancreatic cancer stem cells, which should play an important role in gemcitabine resistance, we subcultured Panc-1 pancreatic cancer cells in increasing concentrations of gemcitabine to generate gemcitabine-resistant Panc-1-GR1 cells. The increase of the proportion of CD133^+^ cells in Panc-1-GR1 cells indicated the enrichment of pancreatic cancer stem cells in gemcitabine-resistant cells. The inhibition of Panc-1-GR1 cell proliferation and the decrease in the proportion of CD133^+^ cells by metformin confirmed its potential to increase sensitivity of pancreatic cancer to gemcitabine. Previously, we established a pancreatic cancer stem cell sphere-formation assay^26^. Sphere-formation is an index of the self-renewal of cancer stem-like cells. This assay was used to test the effect of metformin on Panc-1-GR1 cells. The self-renewal capacity of gemcitabine-resistant pancreatic cancer cells was significantly reduced by metformin treatment. The xenograft experiments using *nu*/*nu* mice also confirmed our findings.

Although metformin has been widely used to treat diabetes for decades, the molecular mechanism of its actions has not been elucidated completely. Most studies suggest that metformin acts through its effect on mTOR and MAPK signaling activation for anticancer actions in pancreatic cancer. Metformin inhibits mTOR and MAPK activation through both AMPK dependent^27^,^28^ and AMPK independent^29^,^30^,^31^ pathways. AMPK-induced activation of p53^32^ and deregulation of miRNAs^33^,^34^ represent other potential mechanisms of action of metformin in pancreatic cancer. In addition, many studies suggest that metformin targets pancreatic cancer stem cells, but whether mTOR or MAPK inhibition mediates the action is still controversial^35^,^36^,^37^,^38^. P70S6K is one of the two major direct targets of mTOR signaling that control the *de novo* pyrimidine synthesis and cell proliferation^13^. P70S6K signaling is also regulated by MAPK/ERK signaling^39^,^40^, and its phosphorylation has been documented to be an independent prognosticator for patients with esophageal squamous cell carcinoma recently^41^.

We analyzed the cancer genome atlas (TCGA) data of the RPPA. Results showed that phospho-P70S6K is one of the four molecules that are associated with the histological grade of pancreatic cancer. We also analyzed the relationship between histological grade of the tumor and P70S6K phosphorylation in pancreatic cancer samples form Union Hospital, Wuhan. The results also showed that P70S6K activation is associated with the histological grade of pancreatic cancer, which confirmed the result of RPPA. These results suggested that P70S6K signaling plays an important role in pancreatic malignancy. However, it must be pointed out that the *P* value for phospho-P70S6K (P = 0.0495) is close to 0.05, which made the result not very convincing. This may have resulted from the small sample size. We next analyzed the role of metformin on phosphorylation of P70S6K when pancreatic cancer is treated with gemcitabine. The results indicated that metformin attenuates the phosphorylation of P70S6K in pancreatic cancer, which was enhanced by gemcitabine treatment. The mechanism of this effect may be associated with the following observations: 1) metformin is a known AMPK activator^42^; 2) metformin inhibits mTOR activation by AMPK-dependent and AMPK-independent pathways in different cancers^27^,^29^,^30^,^43^,^44^,^45^,^46^; 3) ERK signaling plays important roles in chemoresistance^47^,^48^,^49^; 4) gemcitabine activates ERK signaling pathway in pancreatic cancer cells^50^; 5) metformin inhibits phosphorylation of ERK in CD133^+^ pancreatic cancer cells^12^; 6) ERK signaling regulates P70S6K signaling in hepatic and cervical carcinoma cells^39^,^40^; and 7) the RPPA data indicated an association between ERK phosphorylation and P70S6K activation in pancreatic cancer (Fig. 4B). Therefore, we conducted western blotting to analyze the effect of metformin on AMPK and ERK phosphorylation, and conducted RNA interference to study the role of ERK phosphorylation on P70S6K activation and anticancer actions in gemcitabine-resistant pancreatic cancer cells. The results indicated that inhibition of ERK phosphorylation by metformin is at least partly mediated the inhibition of P70S6K activation and increase of sensitivity to gemcitabine in pancreatic cancer cells by attenuating CD133^+^ cells.

mTOR signaling has emerged as a target for therapy; e also conducted a retrospective study in patients of pancreatic cancer with diabeteshowever, most clinical trials of mTOR inhibitors have been disappointing, which may be because mTOR mediated potent negative feedback loops. Carracedo *et al.* observed that mTOR signaling inhibition leads to ERK activation, which attenuates its action in multi type cancers, and recommend a combined therapeutic approach with mTOR and ERK signaling inhibitors^51^. This feedback was also present in pancreatic cancer^52^. Therefore, the mechanism of metformin’s inhibition of P70S6K signaling activation that is mediated by inhibiting ERK phosphorylation in gemcitabine-resistant pancreatic cancer cells indicates the potential of metformin for clinical use. We also conducted a retrospective study in patients of pancreatic cancer with diabetes who underwent pancreatic cancer resection. We observed that the median survival time was 1 month longer and the mean survival time was 4.1 months longer in patients treated with metformin than in those not treated with metformin. Although not statistically significant, metformin showed a trend of beneficial effect, which confirmed the *in vitro* and *in vivo* studies. Very recently, a Korean study on 183 cases of pancreatic cancer with diabetes also showed that metformin benefits patients with pancreatic cancer undergoing chemotherapy; these results were consistant with our study^53^.

In summary, we obtained experimental and clinical clues concerning the potential role of metformin in increasing sensitivity of pancreatic cancer to gemcitabine. The mechanism involves, at least in part, the inhibition of CD133^+^ cells proliferation and suppression of P70S6K activation that mediated by inhibiting ERK phosphorylation. The strength of this study is that the experimental findings are consistent with the clinical findings. The limitations of the study are its retrospective design, the associated bias and the small number of samples in the study in pancreatic cancer patients with diabetes who underwent pancreatic cancer resection. It has been documented that whether patients benefit from mTOR inhibitor depends on the status of mTOR activation in patients with pancreatic cancer^54^, and P70S6K has been documented to be the major direct target of mTORC1 that mediates the anticancer actions of mTOR inhibitors^14^. Thus, patients with P70S6K signaling activation should benefit more from metformin. However, the sample number of our clinical retrospective study hampered stratification by P70S6K singling activation. The findings from our study need to be confirmed in other stratified retrospective studies with sufficient samples and in prospective studies.

## Materials and Methods

### Ethics statement

The study involving human participants was approved by the Ethics Committee of Union Hospital, Huazhong University of Science and Technology (HUST), and informed consents were obtained from all subjects. The methods were carried out in accordance with the approved guidelines. All clinical research was performed on the basis of the principles expressed in the Declaration of Helsinki.

The animal study was approved by the Committee on the Ethics of Animal Experiments of the Union Hospital, HUST. The methods were carried out in accordance with the approved guidelines of the Committee on the Ethics of Animal Experiments of the Union Hospital, HUST.

### Cell culture

We obtained AsPC-1, SW1990 and Panc-1 pancreatic cancer cells from the American Type Culture Collection. Panc-1-GR1 cells are gemcitabine-resistant cells that were generated from Panc-1 cells by subculturing through incremental increases in gemcitabine concentrations, from 0.1 to 1 μM, for 8 weeks. All cell types are K-ras mutated. All cell types were grown in Dulbecco’s modified Eagle medium (DMEM) (Invitrogen, Carlsbad, CA, USA) supplemented 10% fetal bovine serum (FBS) (Gibco, Billings, MT, USA) and penicillin/streptomycin (Invitrogen) at 37 °C with 5% CO_2_. For cancer stem cell spheres culture, Panc-1-GR1 cells were plated in serum-free medium (SFM). The SFM was DMEM-F12 supplemented with 10 ng/mL fibroblast growth factor, 20 ng/mL epidermal growth factor, 5 μg/mL insulin, 2.75 mg/mL transferrin, 2.75 ng/mL selenium (insulin-transferrin-selenium solution) and 0.4% bovine serum albumin.

### Tumor samples and clinical data

The 136 pancreatic tumor samples were all from primary tumors. The samples were from the sample library of Pancreatic Disease Institute, Union Hospital, Wuhan. Follow-up data of 104 patients with pancreatic cancer were from the clinical database of Pancreatic Disease Institute, Union Hospital, Wuhan.

### The reverse phase protein array data

The RPPA data for pancreatic cancer were downloaded from TCGA Research Network: http://cancergenome.nih.gov/. Both clinical and protein expression were retrieved from the datasets. We used log2 transformation (normalized count) for the analysis.

### Immunohistochemistry

Sections (4 μm) were prepared from the paraffin-embedded human primary tumors. Immunohistochemistry was performed following standard procedures. The anti-phospho- P70S6Kantibodies were purchased from Abcam, Shanghai, China. Phospho-P70S6K expression was scored semi-quantitatively on the basis of the percentage of positive cells. Samples with less than 20% positive cells were considered to be weakly to moderately positive, while those with more than 20% positive cells were considered to be strongly positive.

### Quantitative real-time RT-PCR

Total RNA was extracted from AsPC-1 and SW1990 pancreatic cancer cells using TRIzol reagent (Invitrogen) and reverse transcribed using a SuperScript VILO cDNA Synthesis Kit (Invitrogen). The expression of *c-Met*, *Sox2, Oct4* and *β-actin* were quantified using the quantitative SYBR Green PCR kit (TaKaRa Bio, Kusatsu, Shiga, JP), according to the manufacturer’s protocol.

### Flow cytometry

For surface marker detection, cells were resuspended in 100 μL Hank’s balanced salt solution with 1% FBS (Gibco). For isolation of CD133^+^ cells for western blot analysis, cells were resuspended in 100 μL Hank’s balanced salt solution with 1% FBS. Fc Receptor Binding Inhibitor (eBioscience, Inc., San Diego, CA, USA) was added and the sample was incubated for 5 min at 4 °C. After two washes, Anti-CD133 fluorescein isothiocyanate (FITC) (Biorbyt, Cambridge, UK), Anti-CD24 FITC (eBioscience), Anti-CD44 PE-Cy5 (eBioscience) or Anti-ESA PE (eBioscience) were added and the sample was incubated for 30 min at 4 °C. After two washes, the proportions of subpopulation cells that expressed the different surface markers were determined using a FACSCalibur system (BD Biosciences, San Jose, CA, USA) and cell sorting of CD133^+^ cells was done using a FACSAria system (BD Biosciences). Side scatter and forward scatter profiles were used to eliminate cell doublets.

### Cell proliferation assay

Cell proliferation assays were conducted using trypan blue and CCK-8, according to the manufacturer’s instructions. Cells were seeded into a 96-well plate and cultured in 100 μL of DMEM supplemented with 10% FBS. After 24 h, the seeded cells were treated with gemcitabine and/or metformin added to the culture medium. For the trypan blue assay, viable cells were counted after trypan blue staining at the indicated time points. For CCK-8 assay, the medium was exchanged for 110 μL DMEM with the CCK-8 reagent at the indicated time points, and the cells were incubated for 2 h. Absorbance was measured for each well at 450 nm using an auto-microplate reader.

### Cell invasion assay

A cell invasion assay was performed in a 24-well Transwell chamber (Corning, Inc., Corning, NY, USA). First, the 8-μm pore polycarbonate membrane insert was coated with 100 μL of Matrigel (BD Biosciences). The chambers were then placed in 24-well plates; 1 × 10^4^ cells in DMEM supplemented with 0.2% FBS were plated into each upper chamber, and DMEM supplemented with 10% FBS was added to the lower chambers. After incubation at 37 °C for 48 h, cells that had invaded into the opposite side of the membrane surface were stained with crystal violet.

### Western blotting

Flow cytometry sorted cells were washed in PBS and resuspended in RIPA buffer, 1 mM PMSF, 1 mM Na_3_VO_4_, and 1 × protease inhibitor cocktail for 3 min on ice. The lysate was centrifuged at 14,000 × g for 15 min at 4 °C, and the supernatant was used for western blotting. Protein lysates were boiled in loading buffer (Beyotime, Jiangsu, China), resolved by electrophoresis on 8% SDS-polyacrylamide gels, and transferred to PVDF membranes (Amersham Pharmacia Biotech, Amersham, UK). Membranes were probed overnight at 4 °C with primary antibodies recognizing ERK1/2, phospho-ERK (Thr202/Tyr204), AMPKα, phospho-AMPKα (Thr172), P70S6K, and phospho-P70S6K (Thr389) (Cell Signaling, Danvers, MA, USA), with GAPDH (Cell Signaling) as the control. Horseradish peroxidase-conjugated IgG (Beyotime) was used to detect specific proteins. Finally, immunodetection was conducted using chemiluminescent substrates (Amersham Pharmacia Biotech).

### Small interfering RNA

An siRNA targeting human ERK1/2 was purchased from Cell Signaling; a scrambled siRNA was used as a negative control (NC). The cells were plated in 24-well plates and transfected using Lipofectamine 2000 (Invitrogen), according to the manufacturer’s instructions.

### Xenograft experiment

Female *nu*/*nu* mice were obtained from the Experimental Animal Center of Union Hospital, Wuhan, China. For each experiment, six-week-old mice were randomly distributed into equal groups (n = 5) that were treated with gemcitabine only or with metformin. Mice were subcutaneously injected with 1 × 10^7^ Panc-1-GR1 pancreatic cancer cells in the left flank. Gemcitabine was then injected intraperitoneally at 100 mg/kg twice weekly. For the gemcitabine with metformin groups, 800 mg/L of metformin was diluted in their drinking water each day. Both the gemcitabine treatment and the metformin treatment began at the time of injection with the pancreatic cancer cells. Mice were sacrificed 4 weeks after they were injected with pancreatic cancer cells. The tumors were measured and tumor volume (V) was calculated according to V = (length × width^2^)/2.

### Statistical analysis

For quantitative real-time RT-PCR, flow cystometry, cell proliferation assay, cell invasion assays, western blotting, small interfering RNA assay and xenograft experiment, all experiments were performed in triplicate. Data from flow cytometry, cell proliferation assay, cell invasion assay, quantitative real-time RT-PCR, western blotting and the xenograft experiments were presented as the mean ± standard deviation, analyzed by one-way analysis of variance and then compared among groups using an unpaired Student’s *t*-test. Immunohistochemical data were compared using a chi-squared test. Data from the reverse phase protein arrays were analyzed using a general linear model. Survival data were analyzed using survival analysis. A significance threshold of *P* < 0.05 was used. Data were analyzed using SPSS v.11 statistical software (SPSS, Inc.).

## Additional Information

**How to cite this article**: Chai, X. *et al.* Metformin Increases Sensitivity of Pancreatic Cancer Cells to Gemcitabine by Reducing CD133^+^ Cell Populations and Suppressing ERK/P70S6K Signaling. *Sci. Rep.* **5**, 14404; doi: 10.1038/srep14404 (2015).

## Supplementary Material

Supplementary Information

## References

[b1] Andriulli A. *et al.* Neoadjuvant/preoperative gemcitabine for patients with localized pancreatic cancer: a meta-analysis of prospective studies. Ann. Surg. Oncol. 19, 1644–1662 (2012). 22012027 10.1245/s10434-011-2110-8

[b2] Davis J. L., Pandalai P. K., Ripley R. T., Langan R. C. & Avital I. Expanding surgical treatment of pancreatic cancer: the role of regional chemotherapy. Pancreas 41, 678–684 (2012). 22695088 10.1097/MPA.0b013e318249955a PMC3375496

[b3] Dragovich T. Is there a case for personalized therapy of pancreatic cancer? Clin. Adv. Hematol. Oncol. 10, 344–345 (2012). 22706549

[b4] Poruk K. E., Firpo M. A., Adler D. G. & Mulvihill S. J. Screening for pancreatic cancer: why, how, and who? Ann. Surg. 257, 17–26 (2013). 22895395 10.1097/SLA.0b013e31825ffbfb PMC4113008

[b5] Siegel R., Naishadham D. & Jemal A. Cancer statistics, 2013. CA Cancer J. Clin. 63, 11–30 (2013). 23335087 10.3322/caac.21166

[b6] Tempero M. A. *et al.* Pancreatic Adenocarcinoma, version 2.2012: featured updates to the NCCN Guidelines. J. Natl. Compr. Canc. Netw. 10, 703–713 (2012). 22679115 10.6004/jnccn.2012.0073 PMC3807091

[b7] Burris H. A. 3rd *et al.* Improvements in survival and clinical benefit with gemcitabine as first-line therapy for patients with advanced pancreas cancer: a randomized trial. J. Clin. Oncol. 15, 2403–2413 (1997). 9196156 10.1200/JCO.1997.15.6.2403

[b8] Grunewald R., Abbruzzese J. L., Tarassoff P. & Plunkett W. Saturation of 2’,2’-difluorodeoxycytidine 5’-triphosphate accumulation by mononuclear cells during a phase I trial of gemcitabine. Cancer Chemother. Pharmacol. 27, 258–262 (1991). 1998982 10.1007/BF00685109

[b9] Ren C., Chen H., Han C., Wang D. & Fu D. Increased plasma microRNA and CD133/CK18-positive cancer cells in the pleural fluid of a pancreatic cancer patient with liver and pleural metastases and correlation with chemoresistance. Oncol. Lett. 4, 691–694 (2012). 23205084 10.3892/ol.2012.798 PMC3506628

[b10] Hayashi T. *et al.* Interferon-alpha modulates the chemosensitivity of CD133-expressing pancreatic cancer cells to gemcitabine. Cancer Sci. 103, 889–896 (2012). 22320450 10.1111/j.1349-7006.2012.02235.x PMC7659312

[b11] Zhang S. N., Huang F. T., Huang Y. J., Zhong W. & Yu Z. Characterization of a cancer stem cell-like side population derived from human pancreatic adenocarcinoma cells. Tumori. 96, 985–992 (2010). 21388063

[b12] Gou S. *et al.* Low concentrations of metformin selectively inhibit CD133(+) cell proliferation in pancreatic cancer and have anticancer action. PLoS One 8, e63969 (2013). 23667692 10.1371/journal.pone.0063969 PMC3648476

[b13] Robitaille A. M. *et al.* Quantitative phosphoproteomics reveal mTORC1 activates *de novo* pyrimidine synthesis. Science 339, 1320–1323 (2013). 23429704 10.1126/science.1228771

[b14] Morran D. C. *et al.* Targeting mTOR dependency in pancreatic cancer. Gut 63, 1481–1489 (2014). 24717934 10.1136/gutjnl-2013-306202 PMC4145424

[b15] Van Laethem J. L. *et al.* Adjuvant gemcitabine alone versus gemcitabine-based chemoradiotherapy after curative resection for pancreatic cancer: a randomized EORTC-40013-22012/FFCD-9203/GERCOR phase II study. J. Clin. Oncol. 28, 4450–4456 (2010). 20837948 10.1200/JCO.2010.30.3446 PMC2988636

[b16] Vermeulen L., Melo F. D. E., Richel D. J. & Medema J. P. The developing cancer stem-cell model: clinical challenges and opportunities. Lancet Oncology 13, E83–E89 (2012). 22300863 10.1016/S1470-2045(11)70257-1

[b17] Fillmore C. M. & Kuperwasser C. Human breast cancer cell lines contain stem-like cells that self-renew, give rise to phenotypically diverse progeny and survive chemotherapy. Breast Cancer Research 10, R25, 10.1186/Bcr1982 (2008). 18366788 PMC2397524

[b18] Ghods A. J. *et al.* Spheres isolated from 9L gliosarcoma rat cell line possess chemoresistant and aggressive cancer stem-like cells. Stem Cells 25, 1645–1653 (2007). 17412894 10.1634/stemcells.2006-0624

[b19] Wurth R. *et al.* Metformin selectively affects human glioblastoma tumor-initiating cell viability: A role for metformin-induced inhibition of Akt. Cell Cycle 12, 145–156 (2013). 23255107 10.4161/cc.23050 PMC3570504

[b20] Janzer A. *et al.* Metformin and phenformin deplete tricarboxylic acid cycle and glycolytic intermediates during cell transformation and NTPs in cancer stem cells. Proc. Natl. Acad. Sci. USA 111, 10574–10579 (2014). 25002509 10.1073/pnas.1409844111 PMC4115496

[b21] Kirpichnikov D., McFarlane S. I. & Sowers J. R. Metformin: an update. Ann. Intern. Med. 137, 25–33 (2002). 12093242 10.7326/0003-4819-137-1-200207020-00009

[b22] Sadeghi N., Abbruzzese J. L., Yeung S. C., Hassan M. & Li D. Metformin use is associated with better survival of diabetic patients with pancreatic cancer. Clin Cancer Res. 18, 2905–2912 (2012). 22465831 10.1158/1078-0432.CCR-11-2994 PMC3381457

[b23] Wolff R. A. Chemoprevention for pancreatic cancer. Int. J. Gastrointest. Cancer 33, 27–41 (2003). 12909736 10.1385/IJGC:33:1:27

[b24] Li D., Yeung S. C., Hassan M. M., Konopleva M. & Abbruzzese J. L. Antidiabetic therapies affect risk of pancreatic cancer. Gastroenterology 137, 482–488 (2009). 19375425 10.1053/j.gastro.2009.04.013 PMC2735093

[b25] Gou S. M. *et al.* Low Concentrations of Metformin Selectively Inhibit CD133(+) Cell Proliferation in Pancreatic Cancer and Have Anticancer Action. Plos One 8, e63969, 10.1371/journal.pone.0063969 (2013). 23667692 PMC3648476

[b26] Gou S. *et al.* Establishment of clonal colony-forming assay for propagation of pancreatic cancer cells with stem cell properties. Pancreas 34, 429–435 (2007). 17446842 10.1097/MPA.0b013e318033f9f4

[b27] Rozengurt E., Sinnett-Smith J. & Kisfalvi K. Crosstalk between insulin/insulin-like growth factor-1 receptors and G protein-coupled receptor signaling systems: a novel target for the antidiabetic drug metformin in pancreatic cancer. Clin. Cancer Res. 16, 2505–2511 (2010). 20388847 10.1158/1078-0432.CCR-09-2229 PMC2862089

[b28] Ming M. *et al.* Dose-Dependent AMPK-Dependent and Independent Mechanisms of Berberine and Metformin Inhibition of mTORC1, ERK, DNA Synthesis and Proliferation in Pancreatic Cancer Cells. PLoS One 9, e114573 (2014). 25493642 10.1371/journal.pone.0114573 PMC4262417

[b29] Kalender A. *et al.* Metformin, independent of AMPK, inhibits mTORC1 in a rag GTPase-dependent manner. Cell Metab. 11, 390–401 (2010). 20444419 10.1016/j.cmet.2010.03.014 PMC3081779

[b30] Ben Sahra I. *et al.* Metformin, independent of AMPK, induces mTOR inhibition and cell-cycle arrest through REDD1. Cancer Res. 71, 4366–4372 (2011). 21540236 10.1158/0008-5472.CAN-10-1769

[b31] Malki A. & Youssef A. Antidiabetic drug metformin induces apoptosis in human MCF breast cancer via targeting ERK signaling. Oncol. Res. 19, 275–285 (2011). 21776823 10.3727/096504011x13021877989838

[b32] Jalving M. *et al.* Metformin: taking away the candy for cancer? Eur. J. Cancer 46, 2369–2380 (2010). 20656475 10.1016/j.ejca.2010.06.012

[b33] Bao B. *et al.* Metformin inhibits cell proliferation, migration and invasion by attenuating CSC function mediated by deregulating miRNAs in pancreatic cancer cells. Cancer Prev. Res. (Phila) 5, 355–364 (2012). 22086681 10.1158/1940-6207.CAPR-11-0299 PMC3786260

[b34] Tanaka R., Tomosugi M., Horinaka M., Sowa Y. & Sakai T. Metformin Causes G1-Phase Arrest via Down-Regulation of MiR-221 and Enhances TRAIL Sensitivity through DR5 Up-Regulation in Pancreatic Cancer Cells. PLoS One 10, e0125779 (2015). 25955843 10.1371/journal.pone.0125779 PMC4425682

[b35] Lonardo E. *et al.* Metformin targets the metabolic achilles heel of human pancreatic cancer stem cells. PLoS One 8, e76518 (2013). 24204632 10.1371/journal.pone.0076518 PMC3799760

[b36] Mohammed A. *et al.* Antidiabetic Drug Metformin Prevents Progression of Pancreatic Cancer by Targeting in Part Cancer Stem Cells and mTOR Signaling. Transl. Oncol. 6, 649–659 (2013). 24466367 10.1593/tlo.13556 PMC3890699

[b37] Chen G., Nicula D., Renko K. & Derwahl M. Synergistic anti-proliferative effect of metformin and sorafenib on growth of anaplastic thyroid cancer cells and their stem cells. Oncol. Rep. 33, 1994–2000 (2015). 25683253 10.3892/or.2015.3805

[b38] Ling S. *et al.* Metformin inhibits proliferation and enhances chemosensitivity of intrahepatic cholangiocarcinoma cell lines. Oncol. Rep. 31, 2611–2618 (2014). 24788596 10.3892/or.2014.3151

[b39] Jeong J. H. *et al.* Ascochlorin inhibits growth factor-induced HIF-1alpha activation and tumor-angiogenesis through the suppression of EGFR/ERK/p70S6K signaling pathway in human cervical carcinoma cells. J. Cell Biochem. 113, 1302–1313 (2012). 22109717 10.1002/jcb.24001

[b40] Bessard A., Fremin C., Ezan F., Coutant A. & Baffet G. MEK/ERK-dependent uPAR expression is required for motility via phosphorylation of P70S6K in human hepatocarcinoma cells. J. Cell Physiol. 212, 526–536 (2007). 17427199 10.1002/jcp.21049

[b41] Li S. H. *et al.* Phosphorylated p70S6K expression is an independent prognosticator for patients with esophageal squamous cell carcinoma. Surgery 157, 570–580 (2015). 25726316 10.1016/j.surg.2014.10.014

[b42] Viollet B. *et al.* Cellular and molecular mechanisms of metformin: an overview. Clin. Sci. (Lond) 122, 253–270 (2012). 22117616 10.1042/CS20110386 PMC3398862

[b43] Han G., Gong H., Wang Y., Guo S. & Liu K. AMPK/mTOR-mediated inhibition of survivin partly contributes to metformin-induced apoptosis in human gastric cancer cell. Cancer Biol. Ther. 16, 77–87 (2015). 25456211 10.4161/15384047.2014.987021 PMC4622954

[b44] Nair V. *et al.* Mechanism of metformin-dependent inhibition of mammalian target of rapamycin (mTOR) and Ras activity in pancreatic cancer: role of specificity protein (Sp) transcription factors. J. Biol. Chem. 289, 27692–27701 (2014). 25143389 10.1074/jbc.M114.592576 PMC4183806

[b45] Schuler K. M. *et al.* Antiproliferative and metabolic effects of metformin in a preoperative window clinical trial for endometrial cancer. Cancer Med. 4, 161–173 (2015). 25417601 10.1002/cam4.353 PMC4329001

[b46] Vujic I. *et al.* Metformin and trametinib have synergistic effects on cell viability and tumor growth in NRAS mutant cancer. Oncotarget 6, 969–978 (2015). 25504439 10.18632/oncotarget.2824 PMC4359268

[b47] Han S., Li Z., Master L. M., Master Z. W. & Wu A. Exogenous IGFBP-2 promotes proliferation, invasion, and chemoresistance to temozolomide in glioma cells via the integrin beta1-ERK pathway. Br. J. Cancer 111, 1400–1409 (2014). 25093489 10.1038/bjc.2014.435 PMC4183856

[b48] Liu Y. *et al.* SKI-II reverses the chemoresistance of SGC7901/DDP gastric cancer cells. Oncol. Lett. 8, 367–373 (2014). 24959278 10.3892/ol.2014.2083 PMC4063656

[b49] Fernandez-Fuente G., Mollinedo P., Grande L., Vazquez-Barquero A. & Fernandez-Luna J. L. Culture dimensionality influences the resistance of glioblastoma stem-like cells to multikinase inhibitors. Mol. Cancer. Ther. 13, 1664–1672 (2014). 24723451 10.1158/1535-7163.MCT-13-0854

[b50] Wang M. *et al.* pERK1/2 silencing sensitizes pancreatic cancer BXPC-3 cell to gemcitabine-induced apoptosis via regulating Bax and Bcl-2 expression. World J. Surg. Oncol. 13, 66 (2015). 25880226 10.1186/s12957-015-0451-7 PMC4337256

[b51] Carracedo A. *et al.* Inhibition of mTORC1 leads to MAPK pathway activation through a PI3K-dependent feedback loop in human cancer. J. Clin. Invest. 118, 3065–3074 (2008). 18725988 10.1172/JCI34739 PMC2518073

[b52] Soares H. P., Ni Y., Kisfalvi K., Sinnett-Smith J. & Rozengurt E. Different patterns of Akt and ERK feedback activation in response to rapamycin, active-site mTOR inhibitors and metformin in pancreatic cancer cells. Plos One 8, e57289 (2013). 23437362 10.1371/journal.pone.0057289 PMC3578870

[b53] Choi Y. *et al.* The Impact of Diabetes Mellitus and Metformin Treatment on Survival of Patients with Advanced Pancreatic Cancer Undergoing Chemotherapy. Cancer Res. Treat, 10.4143/crt.2014.292 (2015). PMC4720092 25779362

[b54] Utomo W. K. *et al.* mTOR is a promising therapeutical target in a subpopulation of pancreatic adenocarcinoma. Cancer Lett. 346, 309–317 (2014). 24467966 10.1016/j.canlet.2014.01.014

