# Supplementary material for: Metformin Increases Sensitivity of Pancreatic Cancer Cells to Gemcitabine by Reducing CD133+ Cell Populations and Suppressing ERK/P70S6K Signaling
Source: Sci Rep. 2015 Sep 22;5:14404. doi: 10.1038/srep14404 (PMC4585731; doi:10.1038/srep14404)
Supplement: Supplementary Information [file srep14404-s1.pdf]

Title: Metformin Increases Sensitivity of Pancreatic Cancer Cells to Gemcitabine by Reducing $CD133^{+}$ Cell Populations and Suppressing ERK/P70S6K Signaling

Authors:

Xinqun Chai[1], Hongpeng Chu[1], Xuan Yang[1], Yuanpu Meng[1], Pengfei Shi[2], Shanmiao Gou[3].

[1] Department of Hepatobiliary Surgery, Union Hospital, Tongji Medical College, Huazhong University of Science and Technology.

[2] Department of Breast and Thyroid Surgery, Central Hospital of Wuhan.

[3] Pancreatic Disease Institute, Union Hospital, Tongji Medical College, Huazhong University of Science and Technology.

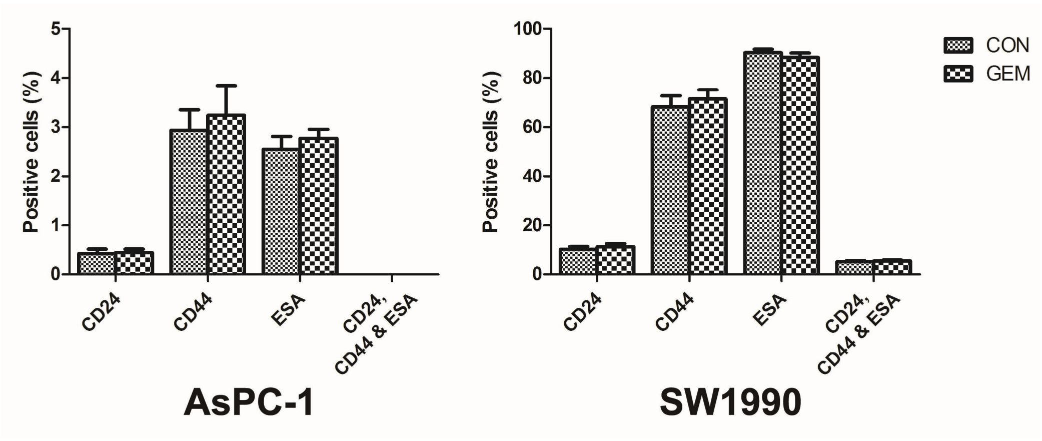

Supplementary Figure S1. Gemcitabine didn't change the proportion of CD24$^+$CD44$^+$ESA$^+$ cells in pancreatic cancer cells. AsPC-1 and SW1990 pancreatic cancer cells were treated with 300 nM gemcitabine for 48 h, and the proportion of CD24$^+$, CD44$^+$, ESA$^+$ and CD24$^+$CD44$^+$ESA$^+$ cells was determined by flow cytometry. Gemcitabine did not affect CD24$^+$, CD44$^+$, ESA$^+$ or CD24$^+$CD44$^+$ESA$^+$ cells (CD24$^+$CD44$^+$ESA$^+$ cells are not detectable among AsPC-1 cells). CON, control; GEM, gemcitabine.

Supplementary Table S1. Effect of gemcitabine on $CD133^+$ and $CD133^-$ pancreatic cancer cells

	Control			Gemcitabine (300 nM, 48 hours)				
	**A**: Cell numbers (relative to control, mean ± standard deviation, %)	**B**: $CD133^+$ cells (mean ± standard deviation, %)	**C**: $CD133^-$ cells (mean ± standard deviation, %)	**D**: Cell numbers (relative to control, mean ± standard deviation, %)	**E**: $CD133^+$ cells (mean ± standard deviation, %)	**F**: $CD133^-$ cells (mean ± standard deviation, %)	**G:** Proportion of survived $CD133^+$ cells ((D*E)/(A*B), %)	**H:** Proportion of survived $CD133^-$ cells ((D*F)/(A*C), %)
AsPC-1	100 ± 2.83	5.18 ± 0.94	94.82 ± 0.94	40.73 ± 2.51	11.00 ± 2.34	89.00 ± 2.34	**86.41**	**38.23**
SW1990	100 ± 2.51	4.38 ± 1.08	95.62 ± 1.08	31.33 ± 1.93	8.40 ± 1.70	91.60 ± 1.70	**60.14**	**30.02**

Supplementary Table S2. Correlation between 193 proteins and histological grade of pancreatic cancer using reverse phase protein array (RPPA)

Protein	CC	95% CI (Lower)	95% CI (Upper)	P	t	df
beta	0.0995	-0.132	0.3207	0.3991	0.8482	72
epsilon	-0.026	-0.253	0.2037	0.826	-0.2207	72
zeta	-0.0996	-0.3208	0.1319	0.3986	-0.8492	72
4E-BP1	-0.1277	-0.3461	0.1039	0.2783	-1.0923	72
4E-BP1_pS65	-0.0936	-0.3153	0.1379	0.4278	-0.7975	72
4E-BP1_pT37_T46	-0.0963	-0.3178	0.1352	0.4143	-0.8211	72
4E-BP1_pT70	0.0694	-0.1616	0.2933	0.5567	0.5906	72
53BP1	0.0697	-0.1614	0.2935	0.5553	0.5926	72
A-Raf_pS299	-0.1267	-0.3452	0.1048	0.2819	-1.0842	72
ACC_pS79	0.1251	-0.1064	0.3438	0.2881	1.0701	72
ACC1	-0.0121	-0.24	0.217	0.9184	-0.1028	72
Acetyl-a-Tubulin-Lys40	0.0495	-0.181	0.2749	0.6752	0.4207	72
ACVRL1	-0.0371	-0.2634	0.193	0.7534	-0.3153	72
ADAR1	0.0071	-0.2217	0.2352	0.952	0.0604	72
Akt	0.1417	-0.0897	0.3586	0.2284	1.2149	72
Akt_pS473	0.0888	-0.1426	0.311	0.4518	0.7564	72
Akt_pT308	0.0161	-0.2132	0.2437	0.8918	0.1365	72
alpha-Catenin	-0.1482	-0.3643	0.0832	0.2077	-1.2712	72
AMPK_alpha	-0.0453	-0.271	0.1851	0.7015	-0.3848	72
AMPK_pT172	0.2365	0.0085	0.4412	0.0425	2.0653	72
Annexin-1	0.0695	-0.1616	0.2933	0.5564	0.5909	72
Annexin_VII	-0.2238	-0.4303	0.0049	0.0552	-1.9489	72
AR	0.0851	-0.1462	0.3076	0.471	0.7247	72
ARHI	-0.1072	-0.3277	0.1243	0.3631	-0.9152	72
ASNS	-0.0378	-0.264	0.1923	0.749	-0.3212	72
ATM	0.1152	-0.1164	0.3348	0.3286	0.9837	72
B-Raf	0.1457	-0.0856	0.3621	0.2155	1.2497	72
Bad_pS112	-0.0224	-0.2496	0.2071	0.8496	-0.1903	72
Bak	0.1118	-0.1197	0.3318	0.3428	0.9549	72
Bap1c-4	0.0735	-0.1576	0.297	0.5337	0.6254	72
Bax	0.04	-0.1903	0.266	0.7354	0.3393	72
Bcl-2	-0.0054	-0.2336	0.2234	0.9635	-0.0459	72
Bcl-xL	-0.0473	-0.2729	0.1831	0.6888	-0.4021	72
Beclin	0.0184	-0.211	0.2459	0.8763	0.1562	72
beta-Catenin	0.0541	-0.1766	0.2791	0.6473	0.4594	72
Bid	0.1336	-0.0979	0.3514	0.2563	1.1442	72
Bim	0.125	-0.1066	0.3437	0.2887	1.0689	72
BRCA2	0.0867	-0.1446	0.3091	0.4625	0.7388	72

c-Jun_pS73	-0.1051	-0.3258	0.1264	0.3729	-0.8967	72
c-Kit	-0.0837	-0.3064	0.1476	0.4782	-0.7129	72
c-Met_pY1235	-0.0802	-0.3032	0.1511	0.4969	-0.6828	72
c-Myc	-0.2441	-0.4477	-0.0166	0.0361	-2.1362	72
C-Raf	0.1563	-0.0749	0.3715	0.1836	1.3428	72
C-Raf_pS338	-0.0187	-0.2462	0.2107	0.8742	-0.1589	72
Caspase-7_cleavedD198	0.1411	-0.0903	0.358	0.2305	1.2092	72
Caveolin-1	0.0583	-0.1725	0.283	0.6219	0.4953	72
CD20	0.1229	-0.1086	0.3418	0.2968	1.051	72
CD31	-0.0708	-0.2946	0.1602	0.5486	-0.6027	72
CD49b	-0.0736	-0.2971	0.1575	0.533	-0.6265	72
CDK1	-0.0896	-0.3117	0.1418	0.4476	-0.7635	72
Chk1	0.186	-0.0444	0.3976	0.1126	1.6062	72
Chk1_pS345	-0.0871	-0.3095	0.1442	0.4603	-0.7423	72
Chk2	-0.137	-0.3544	0.0945	0.2445	-1.1734	72
Chk2_pT68	-0.0824	-0.3052	0.1489	0.485	-0.7019	72
cIAP	0.0614	-0.1695	0.2859	0.6034	0.5219	72
Claudin-7	-0.1579	-0.373	0.0732	0.1789	-1.3573	72
Collagen_VI	0.0278	-0.2019	0.2547	0.8139	0.2363	72
Cyclin_B1	0.1054	-0.1262	0.326	0.3716	0.8991	72
Cyclin_D1	0.124	-0.1075	0.3428	0.2923	1.0608	72
Cyclin_E1	0.0576	-0.1731	0.2824	0.6257	0.4899	72
Cyclin_E2	0.0843	-0.147	0.3069	0.4749	0.7182	72
DJ-1	-0.2208	-0.4277	0.0081	0.0587	-1.9208	72
Dvl3	0.0329	-0.1971	0.2594	0.7808	0.2793	72
E-Cadherin	-0.0309	-0.2576	0.199	0.7935	-0.2627	72
eEF2	-0.0332	-0.2597	0.1968	0.7791	-0.2815	72
eEF2K	-0.0382	-0.2643	0.192	0.7469	-0.324	72
EGFR	0.1643	-0.0667	0.3786	0.1619	1.4134	72
EGFR_pY1068	-0.0328	-0.2593	0.1972	0.7815	-0.2784	72
EGFR_pY1173	0.0618	-0.1691	0.2863	0.6008	0.5256	72
eIF4E	-0.2292	-0.4349	-8.00E-04	0.0495	-1.9982	72
eIF4G	0.0714	-0.1597	0.2951	0.5454	0.6076	72
ER-alpha	0.1886	-0.0417	0.3999	0.1076	1.6294	72
ER-alpha_pS118	0.0934	-0.138	0.3152	0.4285	0.7963	72
ERCC1	0.1383	-0.0931	0.3556	0.24	1.1849	72
ERK2	0.0327	-0.1973	0.2592	0.7823	0.2774	72
ETS-1	0.1862	-0.0442	0.3977	0.1123	1.6077	72
FASN	0.0642	-0.1667	0.2885	0.5867	0.546	72
Fibronectin	0.1393	-0.0921	0.3565	0.2365	1.1936	72
FoxM1	0.0224	-0.2071	0.2497	0.8495	0.1904	72
FOXO3a	-0.1608	-0.3755	0.0703	0.1711	-1.3825	72
FOXO3a_pS318_S321	-0.1412	-0.3582	0.0902	0.23	-1.2106	72
G6PD	-0.0234	-0.2505	0.2062	0.8434	-0.1982	72

Gab2	0.0842	-0.1471	0.3068	0.4758	0.7168	72
GAPDH	0.0393	-0.1909	0.2654	0.7394	0.334	72
GATA3	0.1152	-0.1164	0.3348	0.3286	0.9837	72
GSK3-alpha-beta	-0.0489	-0.2743	0.1816	0.6791	-0.4153	72
GSK3-alpha-beta_pS21_S9	-0.0018	-0.2302	0.2268	0.9876	-0.0156	72
GSK3_pS9	0.0912	-0.1402	0.3132	0.4397	0.7771	72
HER2	0.0602	-0.1706	0.2848	0.6102	0.5121	72
HER2_pY1248	0.0261	-0.2037	0.253	0.8256	0.2212	72
HER3	0.0759	-0.1553	0.2992	0.5204	0.6459	72
HER3_pY1289	0.0299	-0.1999	0.2567	0.8002	0.254	72
Heregulin	0.1615	-0.0695	0.3761	0.1692	1.3888	72
HSP70	0.1496	-0.0817	0.3656	0.2033	1.284	72
IGFBP2	-0.1047	-0.3254	0.1268	0.3746	-0.8935	72
INPP4B	0.0343	-0.1957	0.2607	0.7718	0.2911	72
IRS1	0.1536	-0.0776	0.3691	0.1914	1.3188	72
JNK_pT183_pY185	-0.0196	-0.247	0.2098	0.868	-0.1668	72
JNK2	8.00E-04	-0.2277	0.2293	0.9944	0.007	72
Ku80	0.0887	-0.1427	0.3109	0.4522	0.7559	72
Lck	0.0926	-0.1388	0.3145	0.4326	0.7892	72
LKB1	-0.019	-0.2464	0.2105	0.8726	-0.1609	72
MAPK_pT202_Y204	0.047	-0.1835	0.2726	0.6908	0.3994	72
MEK1	-0.0286	-0.2554	0.2012	0.8088	-0.2429	72
MEK1_pS217_S221	0.0043	-0.2244	0.2326	0.9711	0.0364	72
MIG-6	-2.00E-04	-0.2287	0.2283	0.9984	-0.002	72
Mre11	0.048	-0.1825	0.2735	0.6844	0.4081	72
MSH2	0.1119	-0.1197	0.3319	0.3426	0.9553	72
MSH6	0.1509	-0.0803	0.3668	0.1992	1.2956	72
mTOR	0.0309	-0.199	0.2576	0.794	0.2621	72
mTOR_pS2448	0.0625	-0.1684	0.2869	0.5968	0.5313	72
MYH11	0.0183	-0.2111	0.2457	0.8772	0.155	72
Myosin-IIa-pS1943	-0.0107	-0.2387	0.2183	0.9276	-0.0911	72
N-Cadherin	0.1432	-0.0882	0.3599	0.2235	1.2278	72
N-Ras	0.1115	-0.1201	0.3315	0.3443	0.952	72
NDRG1_pT346	0.0382	-0.192	0.2644	0.7468	0.3241	72
NF-kB-p65_pS536	0.1331	-0.0983	0.351	0.2581	1.1399	72
NF2	0.0088	-0.2201	0.2368	0.9407	0.0747	72
Notch1	0.0599	-0.1709	0.2845	0.612	0.5094	72
P-Cadherin	0.12	-0.1116	0.3392	0.3085	1.0256	72
p21	0.088	-0.1434	0.3102	0.4561	0.7493	72
p27	-0.0203	-0.2477	0.2092	0.8637	-0.1723	72
p27_pT157	-0.0291	-0.2559	0.2007	0.8055	-0.2472	72
p27_pT198	-0.0561	-0.281	0.1747	0.6352	-0.4765	72
p38	0.0706	-0.1605	0.2943	0.5502	0.6003	72
p38_pT180_Y182	-0.0437	-0.2696	0.1866	0.7113	-0.3716	72

p53	-0.0641	-0.2884	0.1669	0.5876	-0.5448	72
p62-LCK-ligand	0.1677	-0.0632	0.3816	0.1532	1.4434	72
p70S6K	0.0966	-0.1348	0.3181	0.4128	0.8238	72
p70S6K_pT389	0.2292	7.00E-04	0.4349	0.0495	1.998	72
p90RSK	0.0554	-0.1753	0.2803	0.6394	0.4706	72
p90RSK_pT359_S363	0.0765	-0.1547	0.2998	0.517	0.6513	72
PAI-1	0.1024	-0.1291	0.3233	0.3853	0.8734	72
Paxillin	0.1432	-0.0882	0.3599	0.2235	1.2278	72
PCNA	-0.0594	-0.2841	0.1714	0.6151	-0.505	72
PDCD4	-0.1865	-0.398	0.0439	0.1116	-1.6106	72
PDK1	0.0211	-0.2084	0.2484	0.8586	0.1788	72
PDK1_pS241	-0.0512	-0.2765	0.1794	0.6646	-0.4353	72
PEA15	-0.133	-0.3508	0.0985	0.2587	-1.1385	72
PEA15_pS116	0.1255	-0.106	0.3441	0.2867	1.0734	72
PI3K-p110-alpha	-0.0105	-0.2384	0.2185	0.9292	-0.0891	72
PI3K-p85	0.0506	-0.18	0.2759	0.6685	0.43	72
PKC-alpha	-0.1195	-0.3388	0.1121	0.3105	-1.0213	72
PKC-alpha_pS657	-0.0436	-0.2694	0.1868	0.7123	-0.3702	72
PKC-delta_pS664	0.0077	-0.2212	0.2358	0.948	0.0654	72
PKC-pan_BetaII_pS660	0.0062	-0.2226	0.2343	0.9583	0.0524	72
PR	-0.0534	-0.2785	0.1772	0.6512	-0.454	72
PRAS40_pT246	0.0379	-0.1923	0.2641	0.7486	0.3217	72
PRDX1	-0.1545	-0.37	0.0767	0.1886	-1.3273	72
PREX1	-0.0277	-0.2546	0.2021	0.815	-0.2349	72
PTEN	0.0412	-0.189	0.2672	0.7272	0.3502	72
Rab11	0.1243	-0.1073	0.343	0.2915	1.0627	72
Rab25	-0.1367	-0.3541	0.0947	0.2455	-1.171	72
Rad50	0.0385	-0.1917	0.2646	0.7448	0.3267	72
Rad51	0.0999	-0.1316	0.3211	0.3971	0.8519	72
Raptor	-0.0169	-0.2444	0.2124	0.8865	-0.1433	72
Rb-M-QC	-0.1813	-0.3935	0.0492	0.1221	-1.5643	72
Rb_pS807_S811	-0.0207	-0.248	0.2088	0.861	-0.1758	72
RBM15	0.102	-0.1295	0.323	0.3872	0.87	72
Rictor	0.1098	-0.1218	0.33	0.3518	0.9372	72
Rictor_pT1135	0.0851	-0.1462	0.3076	0.4709	0.7248	72
S6	-0.064	-0.2883	0.167	0.5882	-0.5439	72
S6_pS235_S236	7.00E-04	-0.2279	0.2291	0.9956	0.0056	72
S6_pS240_S244	0.0354	-0.1947	0.2617	0.7649	0.3002	72
SCD1	-0.209	-0.4176	0.0204	0.0739	-1.8137	72
SF2	-0.0265	-0.2534	0.2032	0.8228	-0.2248	72
Shc_pY317	-0.0874	-0.3097	0.1439	0.4589	-0.7447	72
Smad1	0.0645	-0.1664	0.2888	0.5848	0.5488	72
Smad3	0.1242	-0.1074	0.3429	0.2919	1.0619	72
Smad4	0.042	-0.1883	0.2679	0.7226	0.3564	72

Src	-0.1168	-0.3363	0.1148	0.3219	-0.9975	72
Src_pY416	-0.0894	-0.3115	0.142	0.4487	-0.7617	72
Src_pY527	-0.0362	-0.2625	0.1939	0.7595	-0.3073	72
STAT3_pY705	0.0584	-0.1724	0.2832	0.6209	0.4967	72
STAT5-alpha	0.1285	-0.103	0.3468	0.2752	1.0996	72
Stathmin	0.037	-0.1931	0.2633	0.7544	0.314	72
Syk	-0.0153	-0.2429	0.214	0.8973	-0.1295	72
TAZ	0.1077	-0.1239	0.3281	0.3612	0.9189	72
TIGAR	-9.00E-04	-0.2293	0.2277	0.9942	-0.0073	72
Transglutaminase	0.126	-0.1056	0.3446	0.2849	1.0775	72
TFRC	-0.0169	-0.2445	0.2124	0.886	-0.1438	72
TSC1	0.0496	-0.181	0.275	0.6748	0.4213	72
TTF1	0.1042	-0.1274	0.3249	0.3772	0.8886	72
Tuberin	0.1071	-0.1244	0.3276	0.3635	0.9145	72
Tuberin_pT1462	-0.0174	-0.2449	0.212	0.8833	-0.1473	72
VEGFR2	0.0618	-0.1691	0.2863	0.6008	0.5256	72
VHL	0.0606	-0.1703	0.2851	0.6082	0.5149	72
XBP1	0.0905	-0.1409	0.3125	0.4433	0.7709	72
XRCC1	-0.1005	-0.3216	0.131	0.3943	-0.8571	72
YAP	1.00E-04	-0.2284	0.2286	0.9994	8.00E-04	72
YAP_pS127	-0.0602	-0.2848	0.1707	0.6106	-0.5115	72
YB-1	-0.1665	-0.3806	0.0644	0.1561	-1.4331	72
YB-1_pS102	-0.0415	-0.2675	0.1888	0.7256	-0.3524	72

CC, correlation coefficient; CI, confidence interval; df, degree of freedom.
